# Epidemiology of dengue in a high-income country: a case study in Queensland, Australia

**DOI:** 10.1186/1756-3305-7-379

**Published:** 2014-08-19

**Authors:** Elvina Viennet, Scott A Ritchie, Helen M Faddy, Craig R Williams, David Harley

**Affiliations:** Research School of Population Health, Australian National University, Canberra, ACT 0200 Australia; School of Public Health, Tropical Medicine and Rehabilitative Sciences, James Cook University, P.O. Box 1103, Cairns, QLD 4870 Australia; Research and Development, Australian Red Cross Blood Service, Kelvin Grove, QLD Australia; Sansom Institute for Health Research, University of South Australia, GPO Box 2471, Adelaide, South Australia 5001 Australia

**Keywords:** Dengue, Epidemiology, Delay in notification, Australia, Locally-acquired, Imported cases, Endemic

## Abstract

**Background:**

Australia is one of the few high-income countries where dengue transmission regularly occurs. Dengue is a major health threat in North Queensland (NQ), where the vector *Aedes aegypti* is present. Whether NQ should be considered as a dengue endemic or epidemic region is an ongoing debate. To help address this issue, we analysed the characteristics of locally-acquired (LA) and imported dengue cases in NQ through time and space. We describe the epidemiology of dengue in NQ from 1995 to 2011, to identify areas to target interventions. We also investigated the timeliness of notification and identified high-risk areas.

**Methods:**

Data sets of notified cases and viraemic arrivals from overseas were analysed. We developed a time series based on the LA cases and performed an analysis to capture the relationship between incidence rate and demographic factors. Spatial analysis was used to visualise incidence rates through space and time.

**Results:**

Between 1995 and 2011, 93.9% of reported dengue cases were LA, mainly in the ‘Cairns and Hinterland’ district; 49.7% were males, and the mean age was 38.0 years old. The sources of imported cases (6.1%) were Indonesia (24.6%), Papua New Guinea (23.2%), Thailand (13.4%), East Timor (8.9%) and the Philippines (6.7%), consistent with national data. Travellers importing dengue were predominantly in the age groups 30–34 and 45–49 years old, whereas the age range of patients who acquired dengue locally was larger. The number of LA cases correlated with the number of viraemic importations. Duration of viraemia of public health importance was positively correlated with the delay in notification. Dengue incidence varied over the year and was typically highest in summer and autumn. However, dengue activity has been reported in winter, and a number of outbreaks resulted in transmission year-round.

**Conclusions:**

This study emphasizes the importance of delay in notification and consequent duration of viraemia of public health importance for dengue outbreak duration. It also highlights the need for targeted vector control programmes and surveillance of travellers at airports as well as regularly affected local areas. Given the likely increase in dengue transmission with climate change, endemicity in NQ may become a very real possibility.

**Electronic supplementary material:**

The online version of this article (doi:10.1186/1756-3305-7-379) contains supplementary material, which is available to authorized users.

## Background

Dengue is the most important mosquito-borne viral disease globally [1–3]. The global impact of dengue, recently re-evaluated, is estimated to be 390 million infections per year, which is more than three times the World Health Organization estimate [4]. Dengue affects mainly low to upper middle income countries. Non-immune populations in tropical and sub-tropical countries in Asia, the Pacific, Africa and the Americas are susceptible to large epidemics and endemic transmission of dengue [5]. However, among the 49 high-income countries listed by the World Bank income group [6], six countries are in regions at risk of dengue transmission and Australia is one of them [3, 7]. While not yet considered endemic, dengue case notification occurs throughout the year in Australia. The risk of endemicity is currently restricted to the north of the state of Queensland, where *Aedes aegypti* (L.), the most important vector, is present. Despite increased public health control efforts in North Queensland (NQ), outbreaks have become more frequent over the last two decades. In the near future, larger and more frequent epidemics can be expected, which could result in northern Australia becoming endemic. This threat is mainly potentiated by international travel, domestic mobility and behaviour patterns in NQ. Dengue is imported to Australia via viraemic travellers, mainly from nearby endemic countries from Southeast Asia [8–11]. As a consequence, locally-acquired dengue transmission only occurs in urban areas of NQ from Townsville north through the Torres Strait, where the vector is present. Important outbreaks include the outbreak of 900 cases in Townsville and Charters Towers in 1992–1993, with a duration of 64 weeks [10, 12, 13], over 490 cases in Cairns, Mossman and Port Douglas in 1997–1999 with a 70 week duration [8, 14], multiple outbreaks representing almost 900 cases over a 16-month period (with the exception of two months) in 2003–2004 in Cairns, the Torres Strait Islands and Townsville [10, 15, 16], and over 1000 cases in 2008–2009 in Far North and northern Queensland [17]. Increased public knowledge and the use of molecular diagnostic tools may have increased the number of dengue notifications, but it is unlikely that these factors fully explain the observed increase.

Queensland can be divided into three dengue surveillance areas, i) dengue receptive areas where dengue outbreaks are common, ii) dengue potential areas where vectors are present but contact with viraemic travellers is limited, and iii) dengue-free areas with no recent history of vectors [18]. Dengue surveillance and outbreak responses involve confirmation, notification and management of symptomatic dengue cases. Effective surveillance and notification relies on patients’ and doctors’ awareness of the disease and prompt response to confirmed cases. Clinically suspected cases are required to be notified to the public health units (PHU), whether or not laboratory confirmation is available [19].

We aimed to provide information useful for control programmes and projections of dengue, taking into account demography and viraemic importations. In order to do this we analysed epidemic transmission and propagation spatiotemporally using monthly time series data over 17 years aggregated at the Statistical Local Area (SLA) and Census Collection District (CCD) levels. The use of Geographical Information Systems (GIS) methods enabled the description of epidemic dynamics at the local scale. Here we analyse the periodicity of dengue incidence, develop maps of dengue incidence rates from 1995 to 2011 to assess risk, describe the delay of notification and duration of viraemia of public importance for imported cases and quantify the spread of dengue by *age-group*, *gender*, *year*, *month* and *SLA*.

## Methods

### Study area and study population

**Figure 1:**
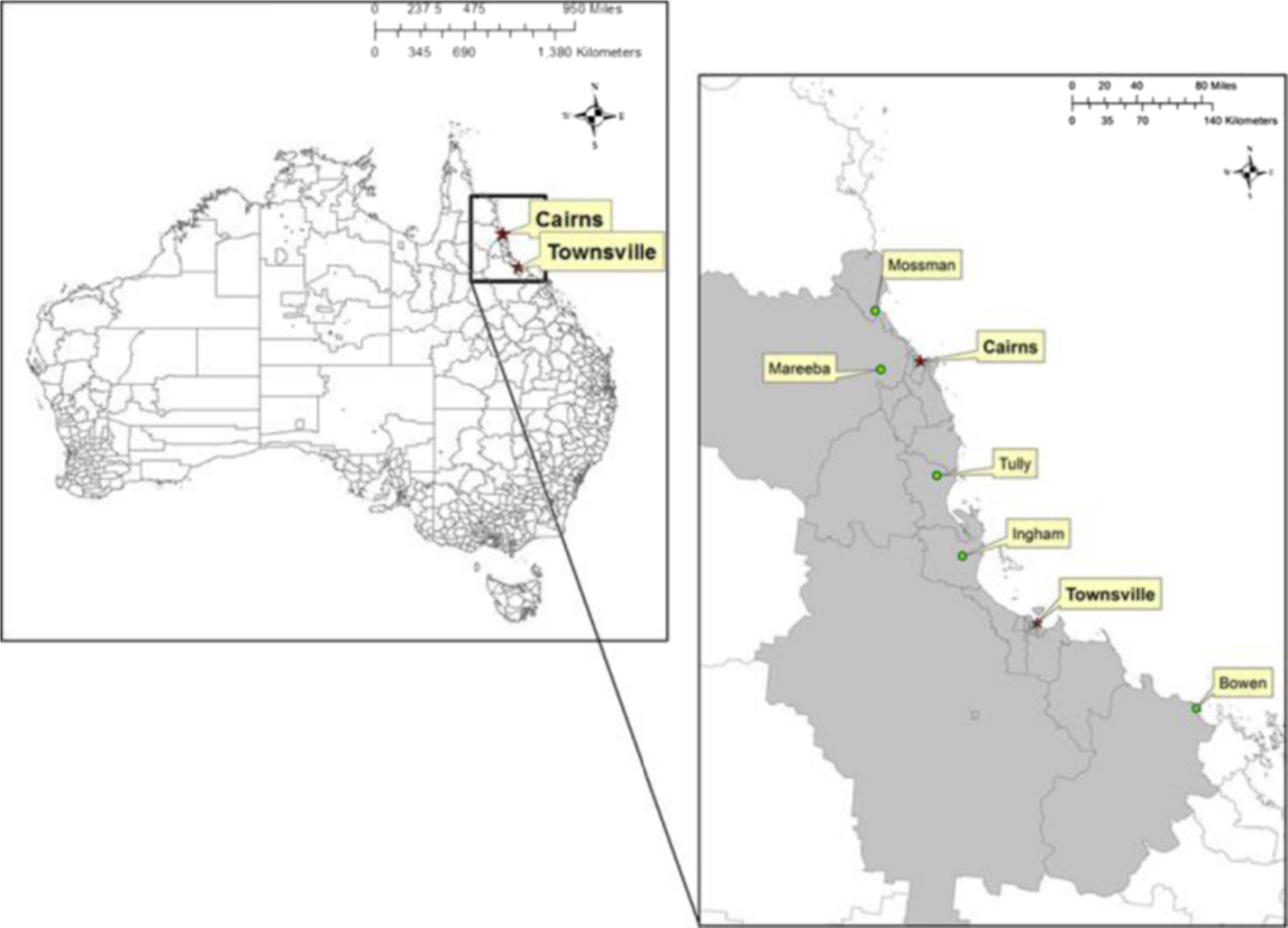
**Map of the study areas (Far North Queensland and North Queensland, Australia).**

De-identified confirmed and probable dengue cases were investigated in Statistical Local areas (SLAs) covering two epidemic areas i) Cairns, South to Tully (S17°56′23″, E145°55′40″) and North to Mossman (S16°27′07″, E145°22’24″) and ii) Townsville, South to Bowen (S20°00′23″, E148°15′15″) and North to Cardwell (S18°15′53″, E146°01′40″), limited by the coast (on the east side) and Mareeba Shire (S) and Dalrymple (S) SLAs boundaries (on the west side) (Figure 1).

Although, dengue outbreaks occurred in the Torres Strait islands in 1996–1997, 2003–2005 [10], the outbreaks were not included in this study because we wanted to consider the transmission in densely populated major centres of NQ rather than in small island communities, disease burden being far greater in the former areas. However, we are aware that the Torres Strait islands are frequently visited from geographically close Papua New Guinea, where dengue cases regularly occur. As a direct consequence, dengue surveillance and control programmes are a priority in the Torres Strait islands [18]. Cairns (S16°55′8.231″, E145°46′14.322″) is located in the tropics of north-eastern Queensland, Australia. Cairns has a tropical monsoon climate characterized by a relatively dry season from June to October and a wet season with tropical monsoons from November to May, with generally mild and dry winters and hotter and more humid summers [20]. The temperature is fairly uniform throughout the year and typical daytime min/max temperature ranges in Cairns are 18°C/26°C in mid-winter and 23°C/31°C in mid-summer. Over the last 30 years (1981–2010), the mean annual rainfall was 1,982 mm with an average number of 155 rain days [21]. On average 71.6% of the mean annual rainfall occurs January to April.

Townsville is also situated on the north-eastern coast of Queensland, approximately 350 km south-east of Cairns and 1360 km North of Brisbane, the state capital in the southeast. Townsville also has a tropical climate, with hot and humid summer months. Monsoon rains continue from late December to early April [22]. Over the last 30 years (1981–2010), the mean annual rainfall was 1,072 mm from 89 rain days [23]. On average 74.1% of the rainfall occurs during December to March. The coolest month is July with daily mean minimum and maximum temperatures of 13.6°C and 25.1°C, respectively. December is the warmest month with daily mean minimum and maximum temperatures of 24.1°C and 31.4°C, respectively [24].

### Ethical consideration and data sources

Following approval of the research protocol by the Australian National University (ANU) Human Research Ethics Committee (HREC number 2012/087) and by Queensland Health Government HREC, daily de-identified data on confirmed and probable dengue cases were provided by the Cairns Public Health Unit, Queensland Health (QH). Demographic data were obtained from the Australian Bureau of Statistics website. Daily meteorological data were retrieved from the Australian Bureau of Meteorology.

### Description of data and case definition

The original dataset (DS1) contained 3187 observations from January 1, 1995 to June 25, 2012, and provided the following information for each case: age at onset, date of onset, gender, residence address, locality name, district description, test type (e.g. IgG, IgM), test result character (equivocal, negative, positive), confirmed case, acquisition place postcode, country of source, and serogroup code. The observations corresponded to either i) confirmed imported cases; ii) confirmed locally-acquired cases; iii) or probable cases, diagnosed clinically (unknown serotype). DS1 was used to calculate the incidence rate based on locally-acquired cases and to develop the time series analysis.

Another data set (DS2) for 1998 to 2011 included cases imported into NQ and FNQ and provided the *age*, *onset date*, *serotype*, *source of importation, delay in notification, date of arrival, place where the case arrived* (primary address), *date of medical consultation and viraemic days of public health importance* in NQ. Dataset 2 was used to summarise the origin of imported cases and analyse information not provided by DS1 (*delay in notification, duration of viraemia*).

The epidemiologically linked cases, called “Epi-linked” cases, are people who cannot be tested or refuse testing, but are closely linked to a case (i.e., live in same premises) and meet case definitions [25].

Dengue is a notifiable disease within Australia. A confirmed case requires clinical evidence and laboratory confirmation. Laboratory methods include virus isolation, nucleic acid testing, detection of dengue non-structural protein 1 (NS1) antigen, dengue virus-specific IgG seroconversion.

A probable case requires clinical (as for a confirmed case), epidemiological (travel or exposure history) and suggestive laboratory evidence (e.g. detection of dengue virus-specific IgM).

The *delay in notification* is defined as the delay between the first medical consultation by the patient with symptoms consistent with dengue fever, and notification to Tropical Public Health Unit (TPHU). However, delay in notification does not necessarily mean that a general practitioner (GP) failed to notify. Indeed, a patient might never consult a GP. The viraemic days of public health importance in NQ corresponds to the duration prior to the implementation of public health measures. The onset of viraemia of public health importance is defined by either the date of onset of symptoms or the date of arrival in the region, whichever came later and the presumed end to viraemia by the date twelve days after either the date of notification to TPHU or if this occurred earlier, date of symptom onset whichever came earlier [26].

Within 24 hours of receiving a notification, public health nurses perform telephone interviews to trace patients, determine their travel history, and determine the date of infection (i.e. date of onset of symptoms minus the intrinsic incubation period (IIP) of 4–7 days), the origin of infection (i.e. imported or locally-acquired dengue), locations visited while viraemic, and the most likely place infection occurred [26]. The IIP is the time between a person being infected and the onset of symptoms due to the infection [27]. When a case is confirmed vector control activities are initiated at and near addresses where a patient spent time while viraemic.

### Data collection and preparation

#### Dengue data

The dataset 1 was cleaned to correct data entry errors, using STATA/IC 12.1 (Additional file 1). We aimed to study the period 1995–2011, thus data for 2012 has been removed (Additional file 2, A). Duplicate records and cases with equivocal and negative test results were excluded from the remaining 3,139 observations (Additional file 2, B). We identified 2,576 individuals, some with missing data including *age, onset date, gender, serogroup code, country of source, locality name, street name or street number* (Additional file 2, C). Addresses were checked via Google Maps™ to identify misspelling in the street name and unrecognised street numbers [28]. Then, longitude and latitude coordinates of each address were obtained using R software 2.15.0 [29]. Corresponding SLA names and CCD codes were added when possible (Additional file 2, D). We determined 17 age-groups using Australian Bureau of Statistics categories with intervals of 5 years for each group (e.g. 0 – 4, 5 – 9, 10 – 14, to 80 years old and over).

#### Demographic data

Demographic data were obtained from the Australian Bureau of Statistics. Population by *age-group* and *gender* at the SLA and CCD levels for 1996, 2001, 2006 and 2011 with linear interpolation for inter-census years were used as the denominator to calculate incidence rates. STATA/IC 12.1 was used to produce appropriate datasets from excel-based Census Community Profiles [30].

#### Incidence rates

Based only on the locally-acquired cases of DS1, the incidence rates were calculated by group year (1995–2004 and 2005–2011) in each SLA and CCD. A time series dataset of number of cases locally-acquired by month and SLA was also developed (Additional file 2, E).

### Data analysis

The objectives of this study are (i) to analyse the epidemiology of dengue in north Queensland to determine if endemicity has occurred; (ii) to identify high risk areas in dengue transmission for targeted prevention and intervention; (iii) to explore the impact of delayed notification on dengue transmission; (iv) to determine the role of imported dengue in local dengue transmission.

We used GIS to visualize the spatial patterns of dengue fever incidence. All geographic layers were processed in a Geographic Information System (ArcGIS 10.1, ESRI, Redlands, CA, USA) using GCS_GDA_1994 Geographic Coordinate Systems, in order to localise cases and aggregate the localisation at the SLA and CCD level to protect each patient’s identity. Therefore, for coordinates, the corresponding SLA name and CCD code were attributed using ArcMap [31] and the Atlas.id websites [32]. The overall age incidence rates are used to visualise the spatio-temporal variation of dengue incidence rate by SLAs, CCDs and grouped into two categories (group-year) 1995–2004, 2005–2011. Thus, we identified SLAs and CCDs (around Cairns) with high standardized incidence rates, in order ultimately to target these for early prevention and vector control. Based on the viraemic imported cases dataset (DS2), we also described notification delays and duration of viraemia.

Using the monthly time series data, we analysed the correlation between the number of imported dengue cases (the independent variable) and the number of locally-acquired cases (the dependent variable), as well as the correlation between the delay in notification (the independent variable) and the size of the outbreak (the dependent variable) (coded 0 for absence of case, 1 for 1 to 10 cases, 2 for 11 to 50 cases, 3 for 51 to 100 cases, 4 for 101 to 200 and 5 for 200 and over). Using the Shapiro-Wilk normality test, we verified that both variables are not normally distributed and we assume that the relation between both variables is not linear [33]. Therefore, we used the Spearman rank correlation method. We assume that if the number of imported cases or the delay in notification increase, the number of locally acquired cases will consequently and logically increase. However, this assumption needed to be verified. We analysed the two largest outbreaks (2003–2004 and 2008–2009). We also performed a cross-correlation analysis to determine the time delay between two time series, with the number of imported dengue cases, the delay in notification, and the duration of viraemia as potential predictors of the number of locally-acquired cases and consequently the size of the outbreak. The maximum (or minimum if negatively correlated) of the cross-correlation function indicates the point in time where the signals are best aligned.

Temporal analyses were performed to describe and quantify the spread of dengue (1995–2011). Graphical representations were used to analyse the distribution of cases by serotype over group-year, as well as to compare case gender and age [34, 35]. For locally-acquired cases, the monthly crude incidence rate was calculated and plotted by district through the study period.

We also performed a Poisson regression analysis to capture the relationship between the incidence rate (outcome) and the exposure variables *age-group*, *gender*, acquisition *month*, acquisition *year* and *SLA*. We hypothesized that *age_group, year, month* and *SLA* would be significant variables. We therefore tested the overall effect of each exposure variable on the outcome variable, using an R function for stepwise Poisson regression (Additional file 3). The AIC statistic was used to identify the best fitting model with the smallest number of parameters. The over-dispersion of the data (underestimation of the variance) was checked using quasi-Poisson regression. The dispersion parameter, indicating over-dispersion (variance greater than the mean), was close to 1 (2.36). Therefore a negative binomial regression model was used and validated by the goodness of fit (1).

1

Then, we estimated the log-rate of dengue cases, the standard error, p-value and confidence interval, and looked for differences in the expected count of dengue cases per person-year, across age-groups, genders, years, months and SLAs. The relative change in the incidence rate for one unit change in any given variable *X*_*i*_ can be estimated by exponentiating its coefficient estimate *β*_*i*_. We expected the highest incidence rates during the hot and wet season, roughly from November to April.

## Results

### Descriptive analysis

A total of 2576 dengue cases were reported in NQ from 1995 to 2011, and 1937 were locally-acquired (LA), 158 were imported (IMP) and the remaining 481 cases (99.2% from 1995–1999) had no origin recorded (NO). On the basis of published data [8, 10, 15, 36, 37], we made the reasonable assumption that those cases were locally-acquired. The absence of origin covered the period 1995–1998, which corresponded to the beginning of the implementation of the dengue case report form. To ensure that this assumption did not influence the final conclusion, we compared epidemiological characteristics between the locally-acquired cases and the ‘no origin recorded’ cases (Table 1). As a sensitivity analysis, we developed two time series datasets (with and without NO cases assumed to be LA) and compared the results. Apart from a higher incidence rate ratio in 1998 (in the data set without NO compared to the dataset with NO cases), we observed similar trends and validated our assumption. Here, we present our analysis on the dataset with NO assumed to be LA. The youngest case was a 4-month-old from Indonesia, and the oldest locally-acquired dengue case was aged 88 years. The mean age of the locally-acquired cases was 38.0 and imported cases were slightly older (40.2). Males constituted 53.5% of cases. The majority of the reported dengue cases were from Cairns and Hinterland district (86.6% of total cases). Finally, 51.8% of the total cases (N = 2576) were infected with DENV-3 (Table 1).

**Table 1:** **Summary of epidemiological characteristics of the 2576 dengue cases in north Queensland, Australia- (Data set 1)**

Variables	Locally-acquired dengue cases	Imported dengue cases	No origin
	N = 1937 *(%)*	N = 158 *(%)*	N = 481 *(%)*
*Age*			
Min	0.7	0.4	1.0
Max	88	74	86.0
Mean	38.1	40.2	37.4
*Gender*			
Female	924 *(47.7)*	61 *(38.6)*	195 *(40.5)*
Male	1010 *(52.1)*	97 *(61.4)*	271 *(56.3)*
NI	3 *(0.2)*	0 *(−)*	*15 (3.1)*
*District*			
Cairns and Hinterland	1651 *(85.2)*	113 *(71.5)*	469 *(97.5)*
Townsville	286 *(14.8)*	45 *(28.5)*	12 *(2.5)*
*Serogroup*			
1	68 *(3.5)*	31 *(19.6)*	2 *(0.4)*
2	535 *(27.6)*	34 (*21.5)*	31 *(6.4)*
3	890 *(45.9)*	27 *(17.1)*	417 *(86.7)*
4	45 *(2.3)*	13 *(8.2)*	0 *(−)*
NI	399 *(20.6)*	53 *(33.5)*	31 *(6.4)*

Major sources of imported cases were Indonesia (24.6%), Papua New Guinea (23.2%), Thailand (13.4%), East Timor (8.9%), and The Philippines (6.7%) (Table 2). The mean annual number of imported dengue cases from 1998 to 2004 and 2005 to 2011 were 9.8 and 23.1, respectively. Importations were mainly in summer (33%) and autumn (38%). The most commonly imported serotype was DENV-2 (34.5%), then DENV-1 (31.0%), DENV-3 (24.4%) and DENV-4 (10.2%).

**Table 2:** **Origin of imported cases, delay in notifications and duration of viraemia (in days) in north Queensland, Australia (Data set 2)**

	Years														
*Source of importation*	1998	1999	2000	2001	2002	2003	2004	2005	2006	2007	2008	2009	2010	2011	Total (%)
Not indicated						3							2		***5 (2.2)***
Cambodia								1	1	2			1		***5 (2.2)***
Cook Islands												3			***3 (1.3)***
East Timor			7	2	2		3	1		1	1	2	1		***20 (8.9)***
Fiji						1						3			***4 (1.8)***
India						1		1				1		1	***4 (1.8)***
Indonesia		1				3				5	7	6	21	12	***55 (24.6)***
Laos										1			1		***2 (0.9)***
Malaysia											1	1	3		***5 (2.2)***
PNG	1	4	6	4	1	8	1	1	1	1	3	1	15	5	***52 (23.2)***
Philippines					1		1	2		2		1	7	1	***15 (6.7)***
Samoa				1							1	1			***3 (1.3)***
Sri Lanka							2	1			2				***5 (2.2)***
Thailand	2	1		3	1			1	1	2		4	9	6	***30 (13.4)***
Vanuatu					2						1	1			***4 (1.8)***
Viet Nam									1		1	2	3	1	***8 (3.6)***
Others^1^											***1***	***1***	***1***	***1***	***4 (1.8)***
*Total*	***3***	***6***	***13***	***10***	***7***	***16***	***7***	***8***	***4***	***14***	***18***	***27***	***64***	***27***	***224***
*Delay of notification* ^*2*^	5.7 [2–11]	5.3 [2–9]	1.8 [0–4]	2.7 [0–8]	1.4 [0–6]	7.3 [0–49]	2.0 [0–6]	3.4 [0–7]	3.2 [1–5]	4.4 [0–10]	6.7 [0–30]	5.4 [0–61]	3.8 [0–23]	3.2 [0–11]	
*Duration of viraemia* ^*2*^	6.7 [3–12]	5.0 [3–9]	4.0 [0–7]	3.9 [0–6]	6.1 [3–11]	7.0 [1–12]	10.0 [5–12]	6.9 [2–12]	6.3 [3–10]	7.3 [0–12]	6.6 [0–12]	8.1 [0–12]	5.2 [0–12]	5.4 [1–12]	

^*1*^ *Others: represent individual source of importation: Guyana, Panama, Timor-Leste and Tonga.*

^*2*^ *Mean and range of delay in notification and duration of viraemia (days).*

**Figure 2:**
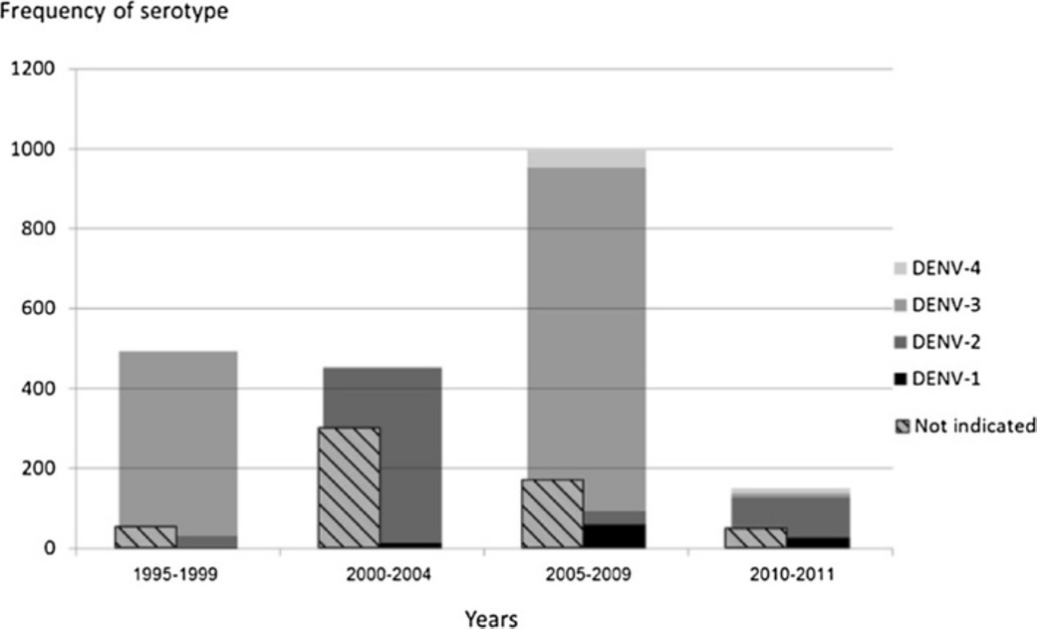
**Frequency of serotype occurrence by group-year in Far North Queensland and northern Queensland, Australia (N = 2576).**

Different s*erotypes* (DENV-1 to DENV-4) predominated in different years (Figure 2). DENV-3 was the most frequent serotype during the *group-years* 1995–1999 and 2005–2009, which corresponded to the 1997–1999 and 2008–2009 outbreaks, respectively, whereas for group-years 2000–2004 and 2010–2011, DENV-2 was commonest. However, since 2005, all four dengue serotypes have co-circulated in NQ.

**Figure 3:**
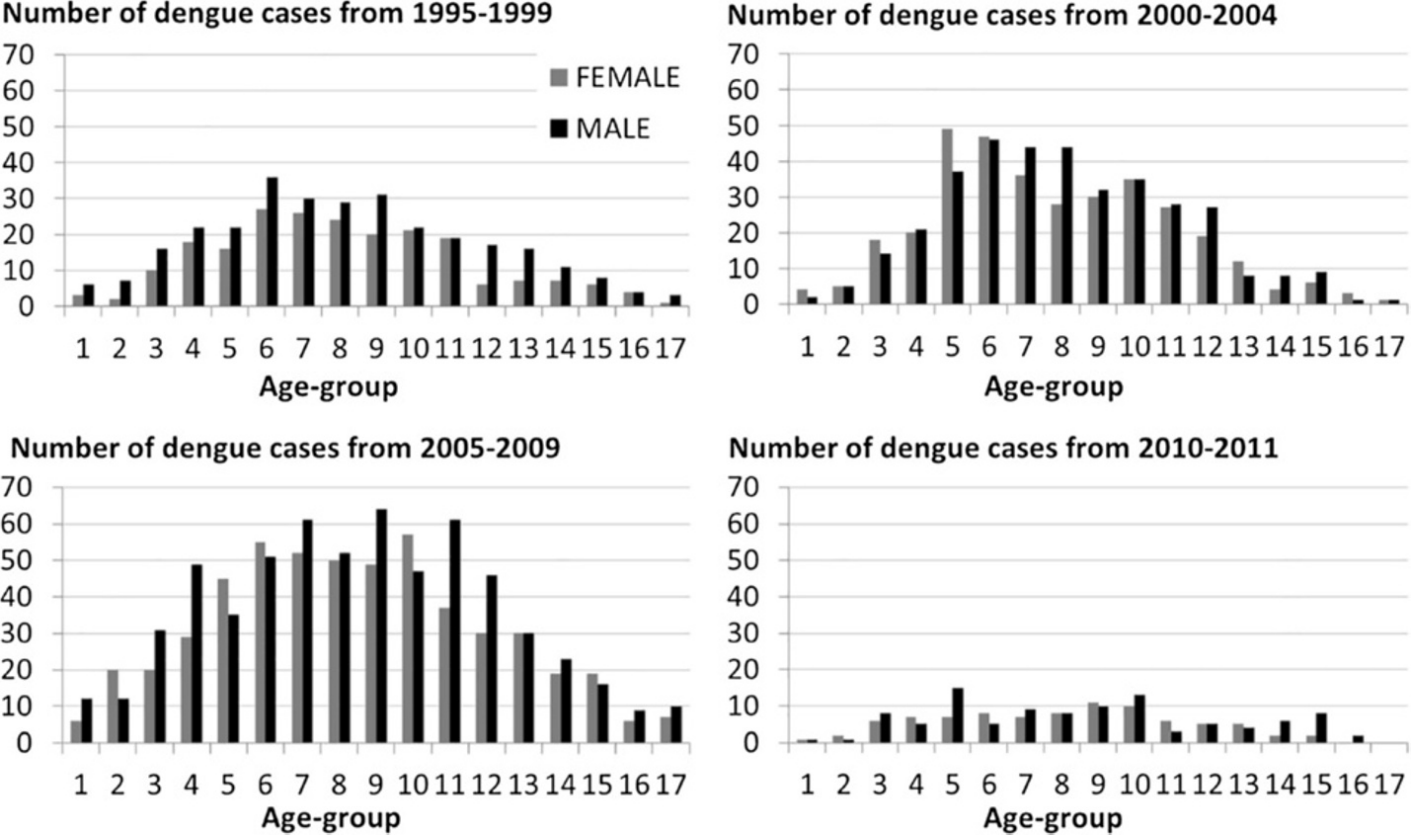
**Dengue case distribution by gender and group-age in Far North Queensland and northern Queensland, Australia (N = 2576).**

The majority were aged 15 to 59 years old with fewer notifications at the extremes of age (Figure 3).

**Figure 4:**
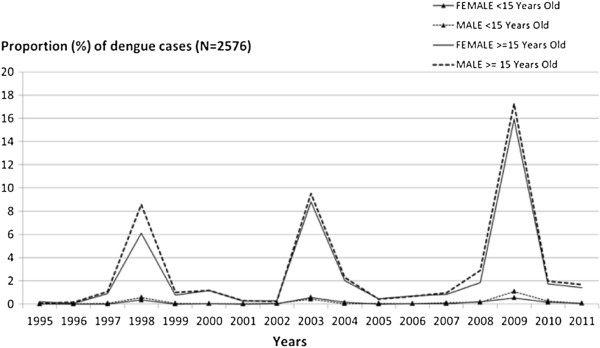
**Proportion of dengue cases in patients < 15 and ≥ 15 years old by gender in North Queensland, Australia (N = 2576).** Patients < 15 years old represented by triangle.

The proportion of notifications for people aged over 15 years increased through time (Figure 4).

### Spatial and temporal distribution

**Figure 5:**
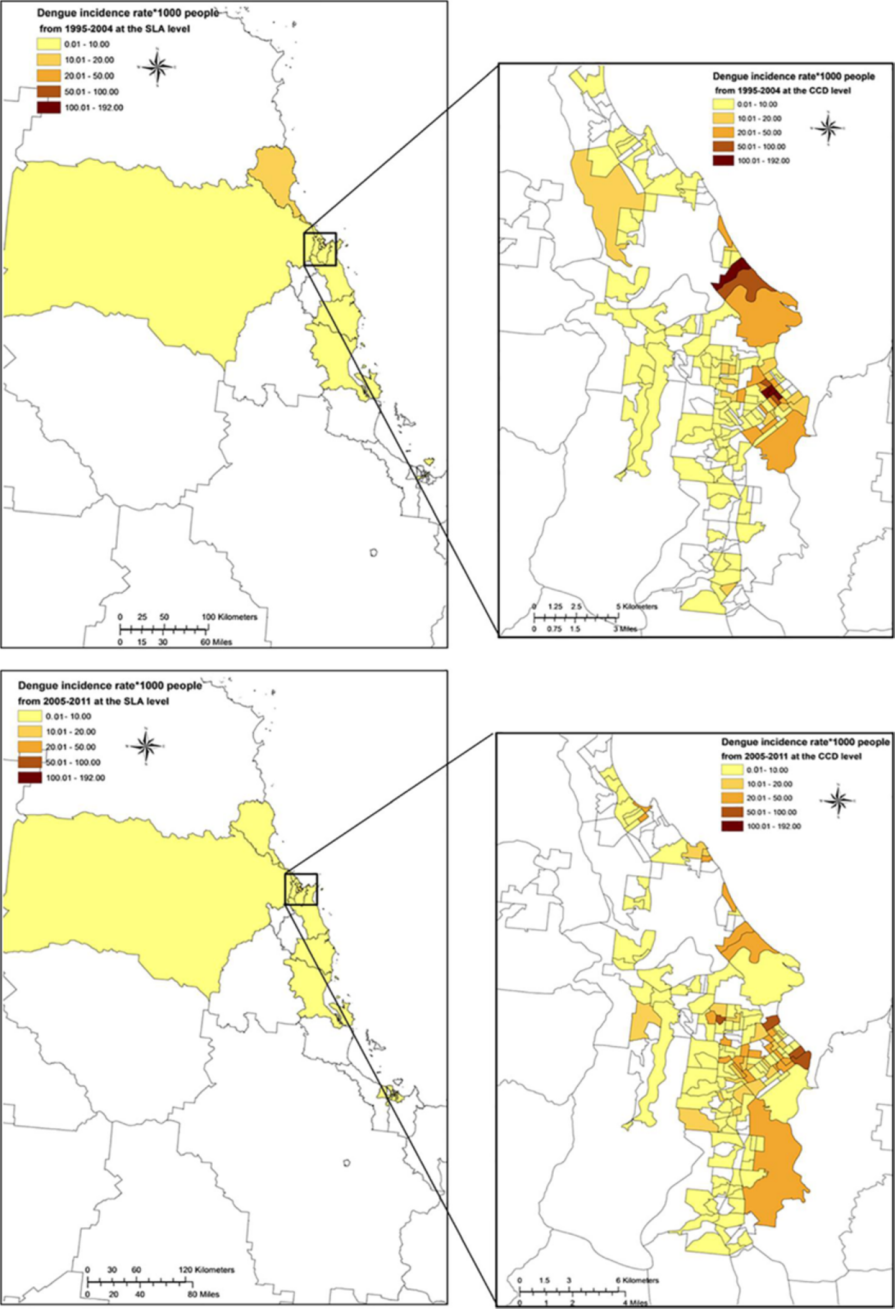
**Maps representing the all age dengue incidence rates by 1,000 people by group of years (1995–2004 and 2005–2011) in North Queensland, Australia.**

Figure 5 presents spatial and temporal distribution for crude incidence rate per 1,000 people by SLA and CCD (around Cairns). The incidence rates were quite low at the SLA level, but in Cairns CCDs ranged from 0 to 192 per 1000 people (from 1995 to 2004), and from 0 to 100 (from 2005 to 2011).

Over the study period, we identified SLAs and CCDs at greater risk including Cairns City, Barron, Central Suburbs, Mt Whitfield, Northern Suburbs, Trinity, Douglas (S), Johnstone (S), North Ward-Castle Hill, and South Townsville. Additional file 4 lists the associated CCDs with an incidence rate above 20 per 1,000 people by group year.

The goodness-of-fit chi-squared test was not statistically significant (p_value = 1.0), so we concluded that the model fitted well. There was no difference in incidence between genders (Table 3). However, *age*, *year*, *month* and *SLA* were significantly associated with age-standardised incidence (p_value < 0.001).

**Table 3:** **Characteristics of locally-acquired dengue cases (N = 2262) in North Queensland, Australia**

Variables	N cases	∑ ***popSLAs***	Rate per 100,000	IRR	95% CI ***(p_value)***	
*Intercept*				0.00	0.00	0.00
*Age*						*(<0.001)*
0-4	33	414,043	7.97	0.19	0.13	0.29
5–9	54	414,353	13.03	0.34	0.24	0.48
10–14	114	410,925	27.74	0.68	0.51	0.90
15–19	158	402,794	39.23	0.89	0.69	1.16
20–24	195	417,016	46.76	0.99	0.77	1.28
25–29	248	430,054	57.66	1.00	0.78	1.28
30–34	226	439,418	51.43	0.99	0.77	1.27
35–39	217	445,533	48.71	1.00	0.78	1.29
40–44 REF	222	435,455	50.98	1.00	-	-
45–49	200	406,998	49.14	1.00	0.78	1.29
50–54	178	363,511	48.97	0.96	0.74	1.24
55–59	136	305,282	44.55	0.80	0.61	1.06
60–64	92	247,990	37.09	0.66	0.49	0.89
65–69	73	195,190	37.39	0.69	0.50	0.95
70–74	67	152,955	43.80	0.82	0.59	1.14
75–79	27	112,084	24.09	0.45	0.29	0.70
80 and over	22	127,418	17.27	0.29	0.18	0.47
*Gender*						*(0.05063)*
Women REF	1066	2,841,472	37.52	1.00	-	-
Men	1196	2,879,547	41.53	1.05	0.94	1.16
*Year*						*(<0.001)*
1995	8	310,427	2.57	0.17	0.08	0.37
1996	1	312,440	0.32	0.02	0.00	0.16
1997	10	314,509	3.17	0.22	0.11	0.44
1998	372	316,547	117.52	8.47	6.08	11.80
1999	42	318,631	13.18	0.86	0.55	1.34
2000 REF	46	320,679	14.34	1.00	-	-
2001	7	322,955	2.17	0.16	0.07	0.37
2002	5	327,675	1.53	0.12	0.05	0.29
2003	461	333,005	138.44	9.04	6.51	12.57
2004	109	338,344	32.22	2.52	1.74	3.64
2005	17	343,688	4.95	0.39	0.22	0.70
2006	34	348,967	9.74	0.80	0.50	1.26
2007	44	353,646	12.44	1.06	0.68	1.63
2008	117	358,125	32.67	2.67	1.86	3.85
2009	851	362,638	234.67	16.06	11.65	22.14
2010	72	367,131	19.61	1.83	1.24	2.71
2011	66	371,612	17.76	1.54	1.04	2.30
*Month*						*(<0.001)*
Jan	319	474,293	61.25	8.55	5.68	12.87
Feb	532	474,866	112.03	0.24	0.10	0.55
Mar	549	475,181	115.53	5.87	3.86	8.94
Apr	252	475,593	52.98	15.84	10.64	23.60
May	154	476,099	32.35	10.81	7.21	16.21
Jun REF	67	476,527	14.06	1.00	-	-
Jul	29	476,945	6.08	2.37	1.50	3.76
Aug	7	477,354	1.47	15.08	10.13	22.45
Sep	35	477,834	7.32	5.57	3.66	8.49
Oct	79	478,328	16.51	2.62	1.67	4.11
Nov	79	478,811	16.49	2.61	1.66	4.09
Dec	160	479,188	33.39	1.23	0.74	2.06

**Figure 6:**
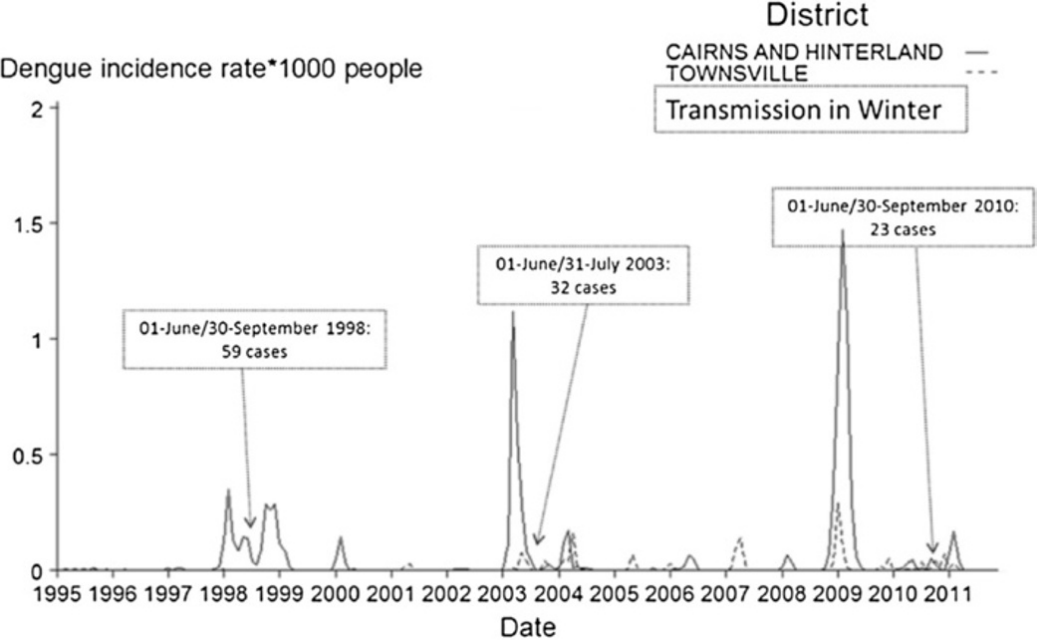
**Incidence rate by 1,000 people by month-year over the study period and by district in North Queensland, Australia (and proof of transmission in winter, number of cases).**

The 20–49 years-old groups had the highest incidence rate ratio (IRR) (Table 3). The youngest (0 to 9 years old) and the oldest age groups (75 and over old) had the lowest IRR. Most cases occurred in March (24.3%), February (23.5%), January (14.1%), and April (11.1%) and only two occurred in August (0.3%) (Table 3). Incidence rates were higher in Cairns and surrounding areas than Townsville (Figure 6). However, transmission also occurred in winter (6.1% of the total cases) (especially in 1998, 2003, 2008, 2009, 2010 and mainly in Cairns with 83% of the cases transmitted in winter).

The analysis concerning the SLAs is presented in Additional file 5. The incidence rate per 100,000 varies from 0.2 (Mt Louisa-Mt St John-Bohle) to 38.89 (Cairns – City). The SLAs with highest incidence were Cairns – City, Barron, Central Suburbs, and Mt Whitfield, Currajong, Douglas (S) and South Townsville. SLAs with high incidence rates are bold in Additional file 5.

### Notification delay and duration of viraemia

**Figure 7:**
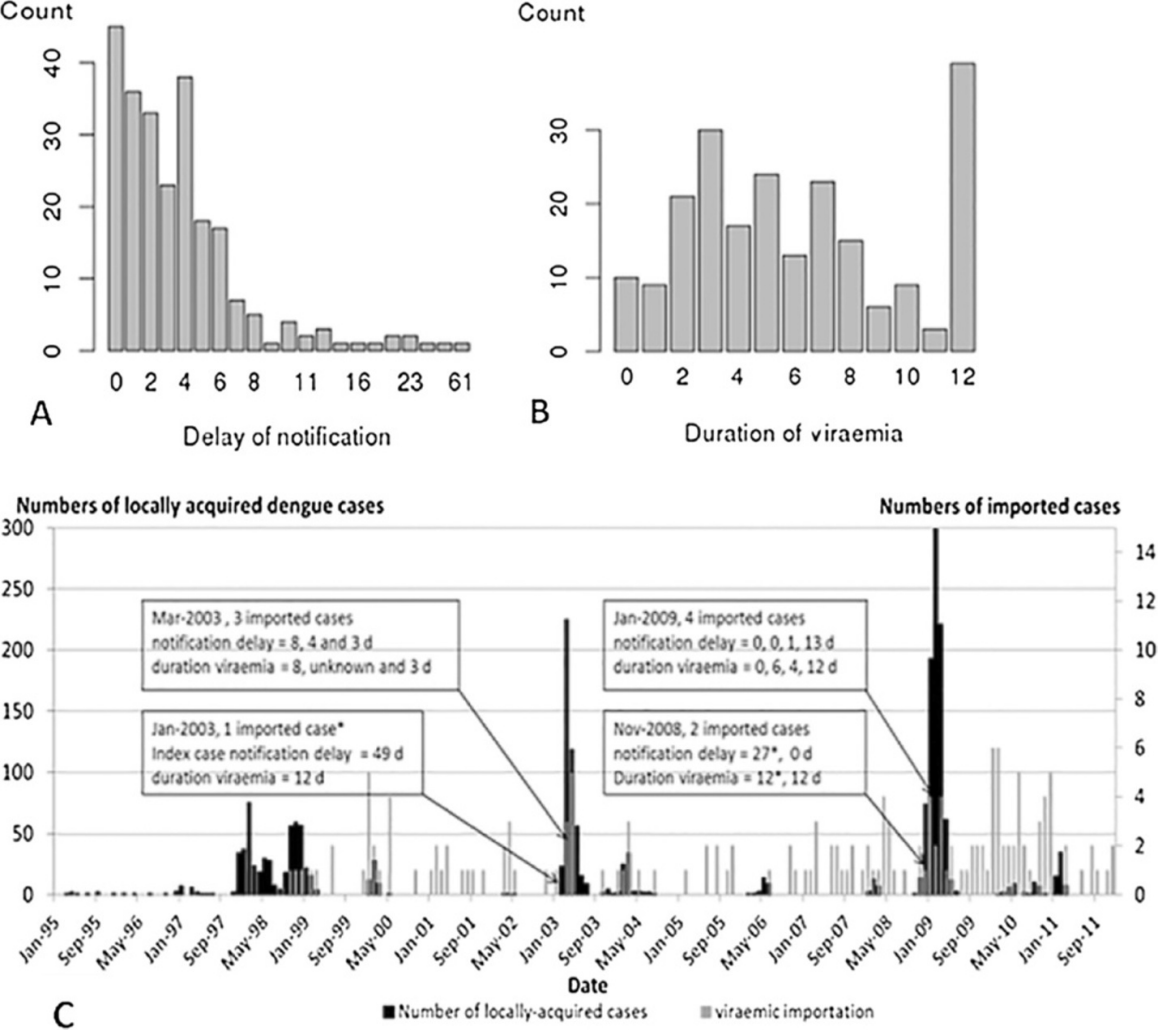
**Characteristics of dengue cases. A**. Delay of notification in days; **B**. Duration of viraemia (days) of public health importance in North Queensland in the imported cases of dengue; **C**. Numbers of dengue cases in Cairns and Hinterland, and information about notification delay and duration of viraemia; *identified as index case.

Notification delay for imported cases ranged from 0 to 61 days (mean of 4.2, median of 3.0 days). Forty-one were notified on the day of consultation (18.3%), 33 within 48 hours (14.7%), seven within 1 week (3.1%), and one each within seven and nine weeks (0.4%) (Figure 7A).

Ninety eight percent (219 cases) of a total of 224 imported cases were provided with information about the duration of viraemia and delay in notification. Twenty-one (10%) of the 219 imported cases were viraemic in Australia, during the two days after the onset of symptoms, twenty-four (11%) in the first five days, and thirty-nine (18%) for 12 days (the maximum possible) (Figure 7B). The highest mean delays in notification were 7.3 [0–49] and 6.7 days [0–30] in 2003 and 2008 with respectively, a mean duration of viraemia of 7 days [1–12] and 6.6 days [0–12] (Table 2). The longest mean duration of viraemia was 10 days [5–12] recorded in 2004, with a mean delay in notifications of 2 days [0–6]. The years 1998, 1999, 2003, 2008 and 2009 were characterized by long mean delay of notification and duration of viraemia (>5 days).

In January 2003, an index case had a notification delay of 49 days with duration of viraemia of 12 days and initiated an outbreak of 459 cases [10, 15] (Figure 7C). In November 2008, at least two viraemic cases were imported into Cairns, with a mean delay of notification of 15 days and a mean duration of viraemia of 12 days. In December 2008, 74 locally-acquired cases were notified, then 193 in January 2009, 299 in February, 221 in March and 62 in April 2009. The mean duration of viraemia of public health importance was 6.1 days [range 0–12 days].

The assumption that the number of locally-acquired cases is correlated with the number of viraemic importations has been confirmed. Indeed, the number of locally-acquired cases increased with the number of imported cases, especially during the 2003–2004 outbreak (in 2003–2004: ρ = 0.91, p_value <0.05; in 2008–2009: ρ = 0.69, p_value <0.05). The duration of viraemia was positively correlated with the delay in notification (in 2003–2004: ρ = 0.65, p_value <0.05; in 2008–2009: ρ = 0.50, p_value <0.05). During the 2003–2004 outbreak, the number of locally-acquired cases was positively correlated with the delay of notification (ρ = 0.48, p_value <0.05). As a consequence during this large outbreak, the size of the outbreak was correlated with the delay of notification (ρ = 0.51, p_value <0.05).

**Figure 8:**
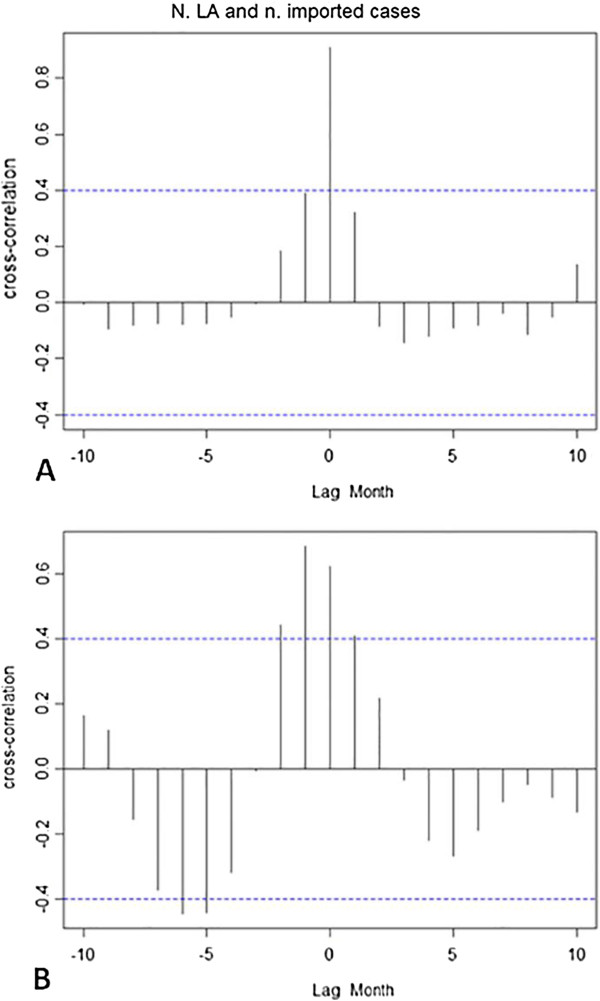
**Cross-correlation analysis between the number of imported dengue cases and locally-acquired dengue cases. A**. during the 2003–2004 outbreak; **B**. during the 2008–2009 outbreak.

The best cross correlation between the number of imported dengue cases and the locally-acquired cases occurred at lag 0 month during the 2003–2004 outbreak (Figure 8A) and at lag 0 to 1 month during the 2008–2009 outbreak. This means that an above average number of imported dengue cases is likely to lead to an above average number of locally-acquired cases during the same month or that immediately following (Figure 8B).

## Discussion

Dengue remains a major public health concern in NQ and may reduce blood donations during outbreaks. We have described the epidemiology of dengue in FNQ and NQ over 17 years and present associations of time, place, and person (age and gender) with incidence. Although this study does not cover the Torres Strait Islands, we carefully reported all cases occurring in NQ, including the 1997–1999 outbreak, omitted by previous work [38]. The existence of clusters in 1995–2004 and 2005–2011 highlights the space and time heterogeneity of dengue determined by environment, weather, human behaviour, and vector distribution, and supports targeted interventions [39]. Notably, whereas several epidemics occurred in the Torres Strait Islands, notably in 1996–1997, 2003–2004 and 2005 (Hanna 2009) (data not provided in our study), few dengue cases have been notified since then. The efficiency of the vector control programme might explain this; however, further studies on the ecology of the vectors and changes in the vector capacity are required. Our study covers a sufficiently long period in FNQ and NQ at a fine scale (CCDs and SLAs) to highlight ‘hot spots’ of increased dengue transmission. To visualise the estimates of incidence rate across FNQ and NQ, we mapped all age incidence rates. The identification of local areas at increased risk defines priority areas for surveillance and control. Hence, despite geographical heterogeneity, we strongly encourage the deployment and maintenance of vector control in all CCDs, but particularly those at high risk.

Imports are a necessary condition for outbreak initiation, but outbreak size is significantly determined by recognition, notification, and public health response. The relation between imported cases in 2010, relative to 2008–9, is illustrative. There were many more imports in 2010, but a far larger outbreak beginning in 2008. While the short extrinsic incubation period of DENV-3 contributed to the latter epidemic, delayed notification and response contributed significantly to outbreak propagation [40]. Notification delay increases epidemic risk [8, 26, 41, 42]. Our study confirms that the epidemics were associated with number of imported cases, and delay in notification, however, further studies using cluster analysis are required. Dengue importations are notified to the TPHU by GPs, public hospitals, private laboratories, and Queensland Health Scientific Services. But as observed, some notifications might be carelessly delayed. The large 2003–2004 and 2008–2009 outbreaks were characterized by index cases with long delay in notification. This resulted from a failure to consult a GP in 2008 and a case of misdiagnosis in 2003 (Ritchie, personal communication). As a consequence, the vector control and public health intervention were delayed, which triggered multiple transmission cycles. Any delay in vector control would increase in the number of human cases and health costs [42]. Thankfully, notification speed and vector control have improved due to increased awareness among local medical practitioners and patients, rapid testing, formation of the Dengue Action Response Team, and development of the Dengue Fever Management Plan [36]. Our study confirms that importations, mainly from Southeast Asia, have increased [43, 44]. The majority of the imported cases were from Indonesia, East Timor, Thailand, The Philippines, and PNG, consistent with national data [44]. Most imported cases were Australian residents returning from overseas [43]. International travel is a necessary cause for dengue transmission in Australia.

Exposure to all four DENV serotypes has increased and raises the probability of severe disease [40, 43]. DENV-3 was the dominant serotype during the 1997–1998 epidemic and 10 years later in 2008 [40], whereas DENV-2 was predominant during the 2003–2004 epidemic and later in 2009–2010. Warrilow *et al*. found that the most commonly imported serotype by travellers from 1990 to 2010 was DENV-1 (39.3%), then DENV-2 (25.7%), DENV-3 (21.4%) and finally DENV-4 (13.6%), originating mainly in Asia, though some imports came from PNG, Pacific Islands and non-Asia-Pacific regions [43]. Consequently, the likelihood that dengue will become endemic is increasing [43], especially if the co-circulation of all four dengue virus serotypes persist in NQ, as has been so since the 1950s in South East Asia [45]. Despite a relatively low incidence of dengue haemorrhagic fever (DHF) and dengue shock syndrome (DSS) in NQ, an increasing rate of secondary or tertiary infections might put Queenslanders at risk for severe dengue. Yet, this direct cause and effect relationship is not verified in Singapore and Haiti, where the incidence of DHF is low despite the endemic co-circulation of DENV [46, 47]. Few dengue-related deaths have occurred in Australia. After the severe 1904–1905 dengue epidemic in Brisbane, Queensland (94 deaths) and the 1925–1926 widespread epidemic in Queensland and New South Wales (NSW) (29 deaths in NSW) [48], a century went by without reported deaths attributed to dengue in Queensland, until two deaths occurred in 2004 and one in 2009 [16, 49].

In most endemic countries, severe dengue occurs generally in young children [46]. However, age for dengue infection has increased in several Southeast Asian countries (Bangladesh, Indonesia, Singapore and Thailand) [50–53]. This is important because age is associated with dengue severity [54–56]. In Queensland, the age-adjusted incidence rate was higher in young adults [25], but severe forms are rare. Incident cases were most common among 25 to 29 year olds, whereas viraemic imports were mostly aged 30 to 49 years.

Males develop dengue more frequently than females in several endemic Western Pacific countries [57–59]. However, in our study we did not find a significant gender difference despite a slight male excess over 15 years old.

Our study confirms that the dengue incidence rate in NQ varies throughout the year with a peak of activity in summer and autumn [38]. At least three epidemics (1997–1999, 2003–2004 and 2010) have been characterized by this pattern. Factors that might explain this trend, include: i) social factors (travel, and consequently viraemic imports being higher in the summer and autumn); and ii) climatic and entomological factors (increased rain and humidity in the wet season from November to April increasing vector breeding and lifespan). In this study, we showed that transmission can also occur during winter, so adult *Ae. aegypti* must be active in winter [48]. Relatively warm daytime temperatures in NQ winters suitable for adult flight and feeding might explain this. Given the likely increase in temperature with climate change, we can expect more transmission during winter. Therefore, endemicity in NQ may become a very real possibility. The space and time heterogeneity of dengue transmission is driven by many interactions between biotic and abiotic factors. Among those factors, heterogeneous biting [60], survival rate [61], both temperature-dependent have important implications for the dynamics of dengue. Blood feeding activity, level of infection, and the extrinsic incubation period (EIP) are temporally mainly driven by weather and virus incursion. The EIP, defined as the period required for the pathogen to develop in the vector and become infective, plays an important role in determining the risk of dengue occurring in a given region. The other important temperature-dependent metric, helping to determine whether an infectious disease can spread through a population or not, is the basic reproduction number *R0*, defined as the number of secondary cases produced by one primary case in a completely susceptible population [62]. The population dynamics (humans and vectors), the timing of introduction relative to the infectiousness of an index case, the contact rate between human and an infected mosquito together with the vector control, socio-economic and cultural factors are also crucial determinants for epidemic propagation [60, 63, 64]. Dengue outbreaks occur annually although dengue cannot yet be considered endemic. Indeed, multiple outbreaks have occurred every year since at least 1991 and throughout the year, especially during the wet season. However, a single serotype has only been transmitted in two consecutive years on three occasions, DENV-3 (1998), DENV-2 (2003) and DENV-2 (2010). In North Queensland dengue transmission requires external input of “imported cases” to create and sustain transmission.

## Conclusions

We report dengue incidence rates by age, year, month, SLA and CCD (Cairns). Based on our findings adult travellers should be targeted for education about dengue. We presented risk areas in NQ and provide further evidence that notification delay is a crucial determinant of epidemic transmission in FNQ. Future research should include analysis of areas with high importation rates and incorporation of demographic, socio-economic, and entomological factors in disease models. Host, vector, and epidemiological factors all contribute to the differences between our Australian data and results from endemic countries. Our study provides empirical evidence regarding two principal public health priorities. These are continued improvement of notification times, and enhanced surveillance for adult travellers from Southeast Asia and PNG. Based on the increasing frequency of dengue outbreaks in NQ and the observation that outbreaks can persist year round, it is essential and timely to reconsider the dengue situation in NQ, strengthen international collaborations and increase awareness of dengue in travellers visiting endemic countries.

## Electronic supplementary material

Additional file 1: **Workflow for analysis.** (PDF 467 KB)

Additional file 2: **Summary of cleaning process and data set exploration (data set 1).** (PDF 263 KB)

Additional file 3: **Poisson regression analysis (stepwise).** (PDF 208 KB)

Additional file 4: **Census Collection Districts and their corresponding Statistical Local Area with a dengue incidence rate > 20 by group year (1995–2004 and 2005–2011) in North Queensland, Australia (based on Figure** 5 **).** (PDF 87 KB)

Additional file 5: **Number and rate of dengue infections among 2262 locally-acquired cases in North Queensland, Australia (resulting from cleaning process E Table** 1 **).** (PDF 252 KB)

## Abbreviations

AIC: Akaike Information Criterion
ANU: The Australian National University
ANOVA: ANalyse Of Variance
CCD: Census Collection District
DENV: Dengue virus
DS1: Dataset 1
DS2: Dataset 2
FNQ: Far North Queensland
GIS: Geographical Information Systems
GP: General practitioner
HREC: Human Research Ethics Committee
IgG: Immunoglobulin G
IgM: Immunoglobulin M
IIP: Intrinsic incubation period
IMP: Imported
IRR: Incidence rate ratio
LA: Locally-acquired
NI: No origin recorded
NQ: North Queensland
NS1: Non-structural protein 1
PHU: Public Health Units
PNG: Papua New Guinea
QH: Queensland Health
S: Shire
SLA: Statistical Local area
TPHU: Tropical Public Health Unit.
