# Supplementary figures and images for: Epidemiology of dengue in a high-income country: a case study in Queensland, Australia
Source: Parasit Vectors. 2014 Aug 19;7:379. doi: 10.1186/1756-3305-7-379 (PMC4261250; doi:10.1186/1756-3305-7-379)

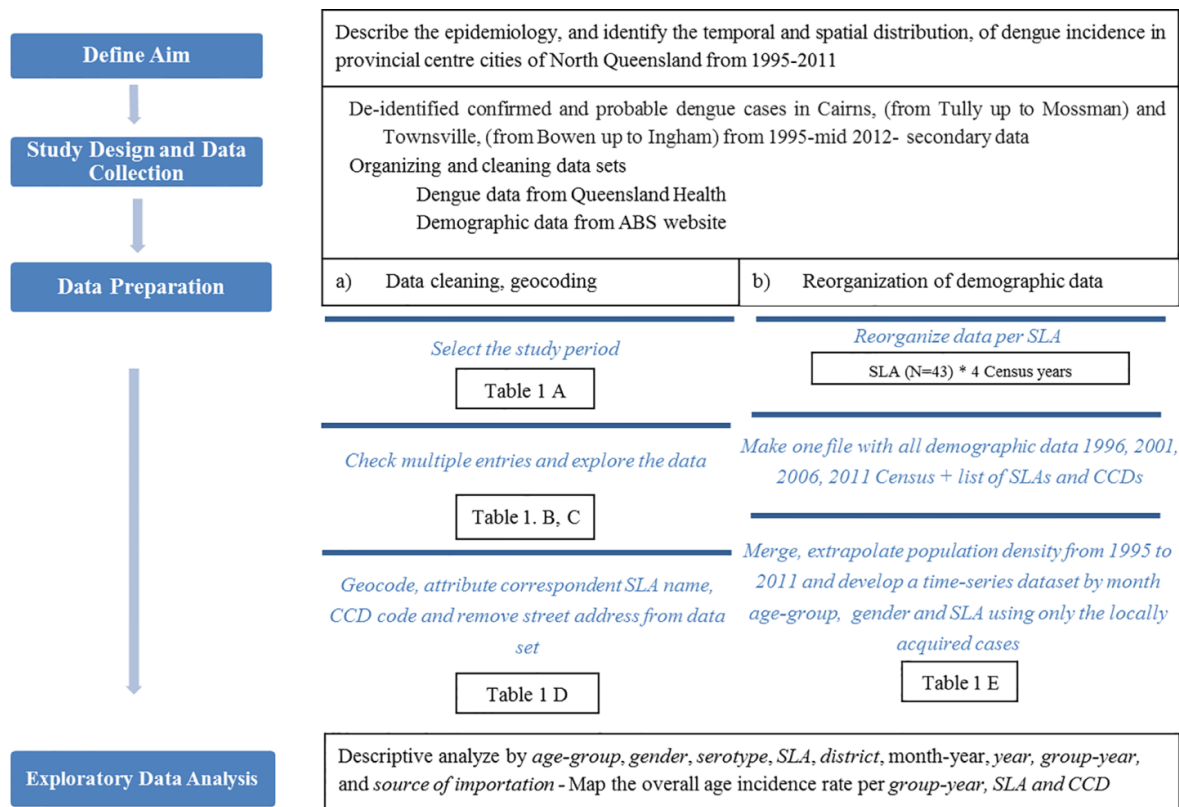

Supplement: Supplementary file 1 — Additional file 1: Workflow for analysis. (PDF 467 KB) [file 13071_2014_1639_MOESM1_ESM.pdf]
