# Supplementary material for: Epidemiology of dengue in a high-income country: a case study in Queensland, Australia
Source: Parasit Vectors. 2014 Aug 19;7:379. doi: 10.1186/1756-3305-7-379 (PMC4261250; doi:10.1186/1756-3305-7-379)
Supplement: Supplementary file 2 — Additional file 2: Summary of cleaning process and data set exploration (data set 1). (PDF 263 KB) [file 13071_2014_1639_MOESM2_ESM.pdf]

Additional file2 Summary of cleaning process and data set exploration (data set 1)

Data set 1	Observations *(%)*	Action
A Cleaning process 1 ***Select the study period***	***N = 3187***	
Year 2012	48 *(1.5)*	removed
B Cleaning process 2 ***Check multiple entries***	***N = 3139***	***saved***
*"*Equivocal*"* and "negative*" Test Results*	532 *(16.9)*	removed
Double entry of individuals	30 *(0.9)*	removed
C Cleaning process 3 ***Information about the data set***	***N = 2576***	***saved***
Epi-linked cases	60 *(1.9)*	kept
Missing *Age*	6 *(0.2)*	kept
Missing *Onset Date*	0 *(-)*	-
"Indeterminate", "not stated/unknown" *Gender*	18 *(0.7)*	kept
Missing *Serogroup code*	483 *(18.8)*	kept
Country of Source "not stated" and "Inadequately described"	481 *(18.7)*	kept
Missing *locality name*	0 *(-)*	-
Misspelling *Street Name*	107 *(4.1)*	corrected
Street Number in the wrong cell	5 *(0.2)*	corrected
Missing *Street Name*	258 *(10.0)*	kept
Missing *Street Number*	309 *(11.9)*	kept
D Cleaning process 4 ***Preparation for Time series data set for locally –acquired cases***	***N = 2576***	***saved*** ***Table 2***
Locally-acquired cases	2418 *(93.9)*	kept
CCD code not attributed	306 *(11.9)*	kept
SLA name not stated or 'Overseas - other"	132 *(5.1)*	removed
"Indeterminate" *Age*	5 *(0.2)*	removed
"Indeterminate" *Gender*	19 *(0.7)*	removed
E Cleaning process 5 ***Develop the time-series dataset by :***	***N= 2262***	
SLA	43	
Age-group	17	
Gender	2	
Year	17	
Month	12	
**Total time-series data set**	***= 298248***	saved and analysed
