# Supplementary material for: Epidemiology of dengue in a high-income country: a case study in Queensland, Australia
Source: Parasit Vectors. 2014 Aug 19;7:379. doi: 10.1186/1756-3305-7-379 (PMC4261250; doi:10.1186/1756-3305-7-379)
Supplement: Supplementary file 3 — Additional file 3: Poisson regression analysis (stepwise). (PDF 208 KB) [file 13071_2014_1639_MOESM3_ESM.pdf]

Additional file 3 Poisson regression analysis (stepwise)

```
fm_pois <- glm(ninf ~ Gender + agegrp + Year + Mon + SLA, family =
poisson(link ="log"), offset= log(popSLA+ 0.5), data = Tab2)

> drop1(fm_pois, test="Chisq")

Single term deletions

Model:
ninf ~ agegrp + Gender + Year + SLA + Mon
       Df Deviance   AIC    LRT Pr(>Chi)
<none>     12246   15786
agegrp 16  12548   16056  302.4   <2e-16 ***
Gender  1  12249   15787    3.8   0.05063 .
Year   16  16418   19926 4172.1   <2e-16 ***
SLA    42  15234   18690 2988.5   <2e-16 ***
Mon    11  14203   17721 1957.7   <2e-16 ***
---
Signif. codes:  0 '***' 0.001 '**' 0.01 '*' 0.05 '.' 0.1 ' ' 1
```
