# Supplementary material for: Epidemiology of dengue in a high-income country: a case study in Queensland, Australia
Source: Parasit Vectors. 2014 Aug 19;7:379. doi: 10.1186/1756-3305-7-379 (PMC4261250; doi:10.1186/1756-3305-7-379)
Supplement: Supplementary file 4 — Additional file 4: Census Collection Districts and their corresponding Statistical Local Area with a dengue incidence rate > 20 by group year (1995–2004 and 2005–2011) in North Queensland, Australia (based on Figure 5 ). (PDF 87 KB) [file 13071_2014_1639_MOESM4_ESM.pdf]

Additional file 4 Census Collection Districts and their corresponding Statistical Local Area with a dengue incidence rate > 20 by group year (1995-2004 and 2005-2011) in North Queensland, Australia (based on figure 5)

CCDs (IR>20) (Fig 5)		Corresponding SLA
**1995-2004**	**2005-2011**	
**3011808**	3011706	Cairns Barron
**3011809**	**3011808**	
**3011810**	**3011809**	
	**3011810**	
3011010	**3011101**	Cairns Central Suburbs
**3011101**	3011109	
3011102	3011114	
3011103	3011305	
3011310	3011403	
3011313	3011502	
3011314	3011503	
3011106	3011006	Cairns City
3011107	3011103	
3011108	3011104	
3011204	3011105	
**3011205**	**3011205**	
3011206	3011211	
3011218	3011212	
	3011213	
	3011214	
	3011215	
3011001	3011406	Cairns Mt Whitfield
	3011408	
-	3011606	Cairns Northern Suburbs
3012205	3012201	Cairns Trinity
	3012705	
3010903	-	Douglas (S)
3010904		
3010914		
-	3012404	Johnstone (S)
	3012405	
	3012407	
3041405	-	North Ward-Castle Hill
-	3042002	South Townsville
	3042004	
	3042005	
