# Supplementary material for: Epidemiology of dengue in a high-income country: a case study in Queensland, Australia
Source: Parasit Vectors. 2014 Aug 19;7:379. doi: 10.1186/1756-3305-7-379 (PMC4261250; doi:10.1186/1756-3305-7-379)
Supplement: Supplementary file 5 — Additional file 5: Number and rate of dengue infections among 2262 locally-acquired cases in North Queensland, Australia (resulting from cleaning process E Table 1 ). (PDF 252 KB) [file 13071_2014_1639_MOESM5_ESM.pdf]

Additional file 5 Number and rate of dengue infections among 2262 locally-acquired cases in North Queensland, Australia (*resulting from cleaning process E Table 1*)

Variables	Cities in Shire	N cases	Mean SLA pop	Rate *100,000	Robust (se)	IRR	95 % CI *(p_value)*	
SLA							*(<0.001)*	
Aitkenvale		4	4900	0.82	0.50	0.03	-4.53	-2.54
**Cairns (C) - Barron**		237	19171	**12.36**	0.12	**0.33**	-1.35	-0.87
**Cairns (C) - Central Suburbs**		457	22043	**20.73**	0.11	**0.68**	-0.61	-0.18
**Cairns (C) – City** REF		430	11056	**38.89**	-	**-**	-	-
**Cairns (C) - Mt Whitfield**		176	11453	**15.37**	0.13	**0.48**	-0.99	-0.48
Cairns (C) - Northern Suburbs		70	16245	4.31	0.17	0.14	-2.29	-1.64
Cairns (C) - Pt B		10	5897	1.7	0.33	0.07	-3.32	-2.04
Cairns (C) - Trinity		148	31744	4.66	0.12	0.15	-2.13	-1.64
Cairns (C) – Western Suburbs		86	11762	7.31	0.15	0.23	-1.75	-1.14
Cardwell (S)	Tully, Innisfail	23	10388	2.21	0.29	0.08	-3.12	-1.99
City		8	2261	3.54	0.36	0.10	-2.99	-1.57
Cranbrook		26	6280	4.14	0.22	0.15	-2.34	-1.46
Currajong		26	2759	9.42	0.23	0.35	-1.52	-0.61
Dalrymple (S)	Dalrymple	1	3785	0.26	0.10	0.00	-19.31	-18.31
Douglas		3	4454	0.67	0.59	0.02	-5.19	-2.89
**Douglas (S)**	Mosmann/Port Douglas	246	13300	**18.5**	0.17	**0.60**	-1.95	-1.28
Garbutt		10	2471	4.05	0.33	0.15	-2.57	-1.28
Gulliver		4	2956	1.35	0.51	0.05	-4.01	-2.01
Heatley		7	4396	1.59	0.39	0.06	-3.59	-2.06
Hermit Park		12	3419	3.51	0.33	0.12	2.76	-1.36
Hyde Park-Mysterton		8	2270	3.52	0.37	0.12	-2.79	-1.36
Johnstone (S)	South Johnstone	87	19252	4.52	0.16	0.17	-2.09	-1.46
Kelso		2	8242	0.24	0.71	0.01	-6.21	-3.41
Kirwan		11	20201	0.54	0.31	0.02	-4.64	-3.43
Magnetic Island		1	2419	0.41	1.01	0.02	-6.14	-2.2
Mareeba (S)	Mareeba	4	18407	0.22	0.58	0.01	-6.28	-4.01
Mt Louisa-Mt St John-Bohle		1	5040	0.2	0.10	0.00	-18.31	-17.91
Mundingburra		15	3861	3.89	0.29	0.14	-2.51	-1.37
Murray		3	9017	0.33	0.58	0.01	-5.67	-3.37
North Ward-Castle Hill		34	5956	5.71	0.20	0.19	-2.05	-1.26
Oonoonba-Idalia-Cluden		2	4891	0.41	0.71	0.01	-5.92	-3.13
Pallarenda-Shelley Beach		2	1004	1.99	0.71	0.08	-3.99	-1.2
Pimlico		2	2440	0.82	0.71	0.03	-4.96	-2.16
Railway Estate		7	2812	2.49	0.39	0.09	-3.23	-1.69
Rosslea		3	1733	1.73	0.58	0.06	-3.94	-1.66
Rowes Bay-Belgian Gardens		18	2493	7.22	0.31	0.24	-2.03	-0.79
**South Townsville**		48	2231	**21.52**	0.19	**0.70**	-0.72	0.01
Stuart-Roseneath		1	1202	0.83	1.00	0.03	-5.65	-1.72
Thuringowa (C) - Pt A Bal		7	18900	0.37	0.39	0.01	-5.21	-369
Vincent		4	2674	1.5	0.51	0.06	-3.89	-1.89
West End		7	3723	1.88	0.39	0.06	-3.55	-2.02
Wulguru		7	4870	1.44	0.42	0.04	-3.94	-2.3
Yarrabah (S)	Yarrabah	4	2392	1.67	0.61	0.06	-3.95	-1.54
